# Romiplostim in Aplastic Anemia: A Single-Center Retrospective Study

**DOI:** 10.7759/cureus.78228

**Published:** 2025-01-30

**Authors:** Vijay Ramanan, Ketki Kelkar

**Affiliations:** 1 BMT Department and Clinical Hematology, Yashoda Hematology Clinic, Ruby Hall Clinic, Pune, IND; 2 Hematology, Anjali Diagnostic Laboratory, Genepath Pathology, Mumbai, IND

**Keywords:** aplastic anemia, bone marrow failure, platelets, romiplostim, thrombopoietin

## Abstract

Background

Acquired aplastic anemia (AA) is an immune-mediated hematopoietic disorder. It is characterized by pancytopenia and hypocellular bone marrow. The first-line treatment approach to AA includes immunosuppressive treatment (IST) using the combination of antithymocyte globulin (ATG) and cyclosporine A. Those patients who do not respond to this first-line treatment or have refractory disease succumb to bleeding or infections within five years following their diagnosis even after IST. Romiplostim, a thrombopoietin (TPO) receptor antagonist, promotes trilineage hematopoiesis in patients with AA. A retrospective study assessed the safety and effectiveness of romiplostim, as monotherapy among patients with refractory AA in Indian settings.

Methods

The case record forms of patients diagnosed with refractory AA and receiving treatment with weekly doses of 250 mcg romiplostim and concomitant medications were reviewed at Yashoda Hematology Clinic, India. The primary outcome was to evaluate the increase in platelet count/percentage and the safety of romiplostim in these patients. The secondary outcomes were a change in total leucocyte count (TLC) and hemoglobin (Hb) levels from baseline after romiplostim therapy.

Results

Data from 28 patients diagnosed with AA and having received romiplostim subcutaneously in a dose of 250 mcg weekly were analyzed. There was a significant improvement in platelet count which increased by 0.064 × 10^9^/L units (95% CI: 0.021-0.107) at a 100-day interval from the initial measurement. The TLCs also increased by 0.022 × 10^9^/L units (95% CI: 0.002-0.042). The mean Hb levels increased from 7.61 gm/L to 13.38 gm/L (95% CI, p < 0.001). No severe adverse events were reported.

Conclusion

Romiplostim demonstrated clinically significant outcomes with a favorable safety profile in patients with refractory AA.

## Introduction

Aplastic anemia (AA) is a severe condition marked by pancytopenia and a reduction in bone marrow cellularity, resulting from the destruction of hematopoietic stem cells by lymphocytes [1-4]. In AA, CD8+ and CD28- cytotoxic T-helper cells become activated at the cellular level, leading to a reduction in cell division and the induction of apoptosis in hematopoietic stem cells [5].

The continuous immune assault and significant reduction of tissue stem cells result in limited improvement in AA. Individuals with AA face critical bleeding because of thrombocytopenia and are at a higher risk of severe infections due to neutropenia and/or exhaustion caused by anemia. Additionally, increased mortality rates are commonly observed in AA, particularly when pancytopenia worsens [5]. Refractory AA presents as the persistence of severe cytopenias at six months after IST. It is a lack of response to first-line immunosuppressive therapy (IST) with antithymocyte globulin (ATG) and cyclosporine [6].

Following IST, over 40% of refractory AA patients succumb to bleeding or infection within five years of being diagnosed [7].

The global incidence rate of AA ranges from 0.7 to 4.1 cases per million people, with similar prevalence among men and women. However, the Asian incidence rate of AA is said to be two to three times higher than that in the West, with an annual incidence of 8.2 per 1,000,000 people [7]. This is attributed to common factors in developing Asian countries, such as lower socio-economic status and increased exposure to pesticides, chemicals, toxins, and pathogens, which are implicated in the development of AA. Given the prevalence of lower socio-economic status in India, it is reasonable to assume a higher incidence of AA [8]. The exact incidence of AA in India is unknown due to a lack of epidemiological studies. However, a hospital-based Indian study reported that 20-40% of pancytopenic patients are diagnosed with AA in referral centers. Additionally, an All India Institute of Medical Sciences (AIIMS) study showed that 200 new Indian patients with AA were identified in the aplastic clinic [9].

Management of AA patients is challenging due to the long-term duration from diagnosis to treatment and the high cost of therapy. Notably, most Indian patients are heavily pre-transfused, usually with leuco-depleted blood products. Moreover, nearly 40% of patients are infected before initiation of any treatment and more than 90% receive IST before stem cell transplantation (SCT) [9].

Hematopoietic SCT and IST are the primary treatment modalities for AA [10]. For patients with severe AA who are not eligible for bone marrow transplantation, IST with ATG plus cyclosporine is the standard of care. However, approximately 33% of the patients who are unable to undergo transplantation and receive IST fail to respond or experience relapse following initial treatment [1]. While some patients may respond to a second course of IST or cyclosporine alone, other pharmacological approaches have generally resulted in unsuccessful responses for patients with refractory disease, although they have been used for salvage therapy [3].

Thrombopoietin (TPO) plays a crucial role in platelet formation. It binds to the TPO receptor (c-MPL; also known as TPO-R) on megakaryocytes; this leads to platelet maturation and release. Hematopoietic stem cells and progenitor cells also express c-MPL on their surface, and the addition of recombinant TPO expands the pool of hematopoietic stem cells in culture, resulting in the recovery of blood counts. In c-MPL-knockout mouse models, a significant decrease in stem cells was observed, in addition to megakaryocytopenia and thrombocytopenia. TPO is commonly used, along with other growth factors and interleukins, to stimulate stem cells in vitro. Furthermore, a homozygous nonsense mutation in the c-MPL gene, MPL, has been found in patients with familial AA. These findings collectively suggest that c-MPL could be a potential therapeutic target for addressing the process of multilineage cell depletion (thrombocytopenia, erythropenia, and neutropenia) associated with AA [3,7].

Romiplostim is categorized as a peptibody, specifically an Fc-peptide fusion protein that acts as a c-MPL agonist. It promotes the production of endogenous TPO, which in turn facilitates the proliferation and differentiation of megakaryocytes within the bone marrow [3]. As a TPO receptor agonist, romiplostim activates intracellular transcriptional pathways through the TPO receptor, thereby stimulating hematopoietic stem and progenitor cells. A phase II clinical trial assessing romiplostim as a standalone treatment demonstrated a positive safety profile and effectiveness in hematological responses in patients with immunosuppressant-refractory AA [7]. Recently, romiplostim has received approval for use in refractory AA in both Korea and Japan, following an international multi-center trial that reported an approximate 83.9% response rate in patients with refractory AA [7]. However, the effectiveness and safety of this medication in real-world scenarios among Indian patients require further investigation. The current study aimed to evaluate the safety and efficacy of romiplostim, in Indian patients suffering from refractory AA.

## Materials and methods

Study design

This was a single-center retrospective study conducted at Yashoda Hematology Clinic, located in India. The Royal Pune Independent Ethics Committee approved the study with IEC number RPIEC020524. The study followed national laws and regulations, the International Conference on Harmonisation of Good Clinical Practice guidelines, and the ethical principles of the Declaration of Helsinki.

Patients

The study comprised 28 patients diagnosed with refractory AA, all aged 24 years or older, who exhibited resistance to treatments such as ATG, cyclosporine, danazol, stanozolol, thalidomide, prednisolone, and eltrombopag. The diagnosis of AA was established through bone marrow and cytogenetic evaluations, and presented with thrombocytopenia, defined as a platelet count of 30 × 10⁹/L or lower. The diagnosis was determined based on bone marrow cellularity, following standard procedures deployed by the hematologists. Data from chart reviews regarding patient demographics and disease characteristics, including age, sex, prior therapies before initiating romiplostim, any concurrent treatments for AA, complete blood counts at diagnosis, prior to and during romiplostim therapy, as well as the dosage and duration of romiplostim, and any adverse events experienced during treatment were meticulously documented.

Dosing regimen

Eligible patients had received romiplostim 250 mcg weekly as the standard dose, along with concomitant medications, namely, danazol, hSP72, cyclosporine A, filgrastim, stanozolol, eltrombopag, prednisone, or thalidomide.

Outcomes

The primary objective was to evaluate the increase in the count/percentage of platelets in patients on romiplostim. The secondary objectives were to evaluate the change in hemoglobin (Hb) levels, total leucocyte count (TLC), and the safety of romiplostim at the end of 12 weeks.

Statistical analysis

Descriptive statistics were performed for demographics, including age, sex, and body weight in terms of frequency and percentages. Stacked graphs were used to describe the numerical values across the dosing pattern with time and number of patients. The efficacy with mean of Hb levels, TLC, and PC with 95% CI < 0.05 was considered statistically significant. A paired t-test was performed with the hypothesis that the increment is positive, and a p-value < 0.05 was considered indicative of statistical significance.

## Results

The study population comprised 28 patients with refractory AA. The baseline demographics and variable period of follow-up as per the number of patients have been listed in Tables 1-2.

**Table 1:** Baseline demographic and clinical characteristics of the study population (n = 28) NA: Not available

Variables	N (%)	Mean (SD)
Males	9 (25%)	NA
Females	19 (75%)	NA
Age (years)	24 (86%)	37.12 (18.27)
Body weight (kg)	28 (100%)	61.36 (14.28)
Platelet count (count/cu.mm)	28 (100%)	70,089.29 (100,204.2)
Hemoglobin level (gm/L)	28 (100%)	7.61 (2.24)
Total leucocyte count (count/cu.mm)	28 (100%)	4,196.11 (3320.09)

**Table 2:** Variable period of follow-up as per number of patients with refractory aplastic anemia NA: Not available

Period	Follow-up period from baseline (days)				
						N (%)	Mean	SD	Median	
Visit 2	27 (96.4%)	160.48	364.2	56	
Visit 3	26 (92.8%)	290.81	438.11	124.5	
Visit 4	21 (75%)	365.33	519.45	203	
Visit 5	17 (60.7%)	525.65	519.12	339	
Visit 6	12 (42.8%)	662.08	585.9	448	
Visit 7	9 (32.1%)	859.89	686.73	556	
Visit 9	6 (21.4%)	980.83	294.4	862	
Visit 11	4 (14.2%)	1034.75	164.51	1044.5	
Visit 13	1 (3.5%)	1391	NA	1391	

The mean age of the patients was 37.12 years (n = 24) with a greater number of females in the study population. The baseline PC was 70,089.29 cu.mm, the Hb level was 7.61 gm/L, and the TLC was 4,196.11 mm^3^. The most common concomitant medication with romiplostim were danazol, eltrombopag, thalidomide, and cyclosporine.

**Figure 1:**
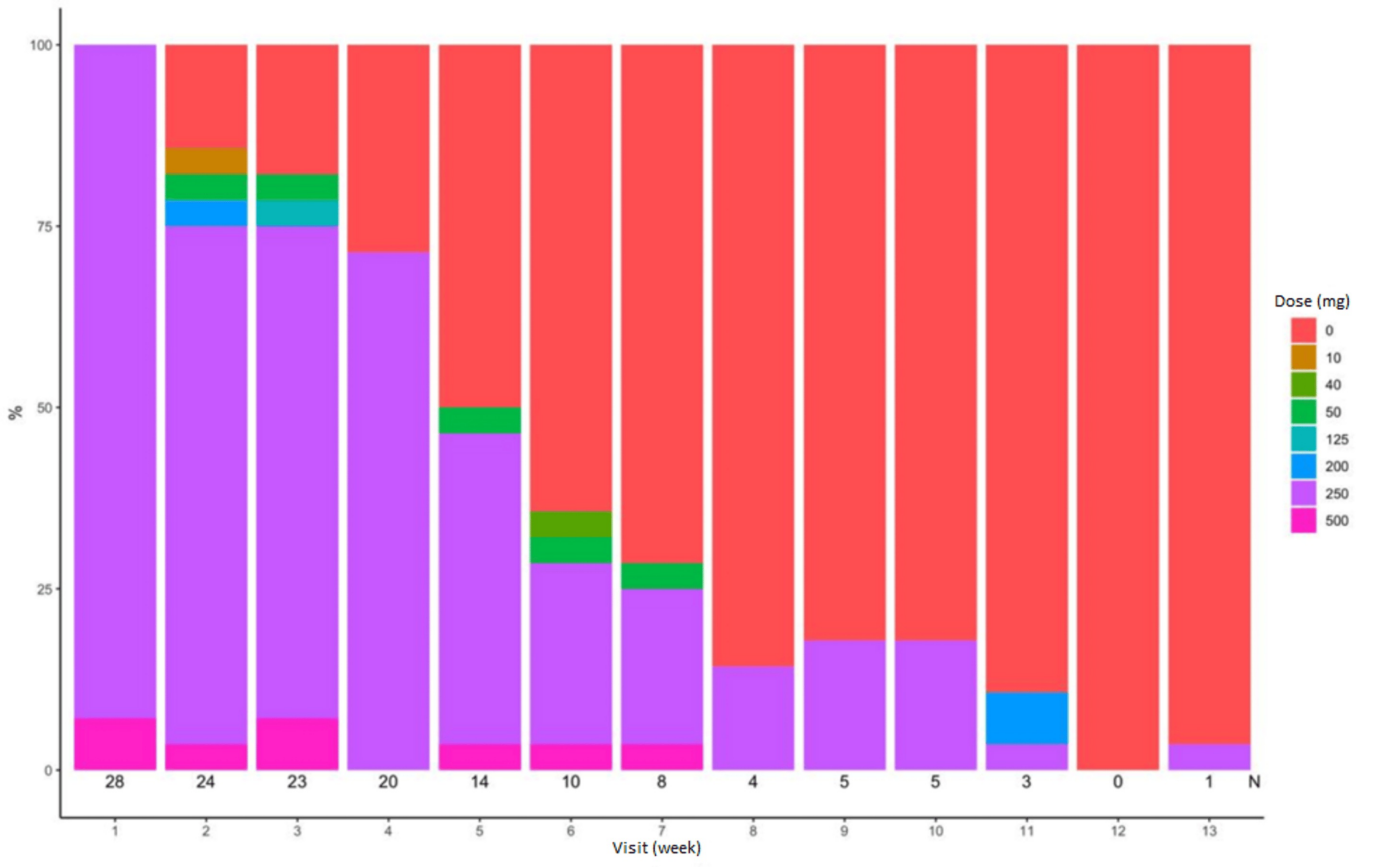
Romiplostim doses administered to the patients across visits

All the patients responded to romiplostim on hematological parameters (platelet count, Hb, and TLC) at variable time periods (Table 2). Figure 1 shows romiplostim doses across the study period.

The chart shows a decrease in patients receiving higher doses of romiplostim (e.g., 500 mcg) over time, with a large proportion ultimately receiving 0 mcg by the later visits.

Efficacy

**Figure 2:**
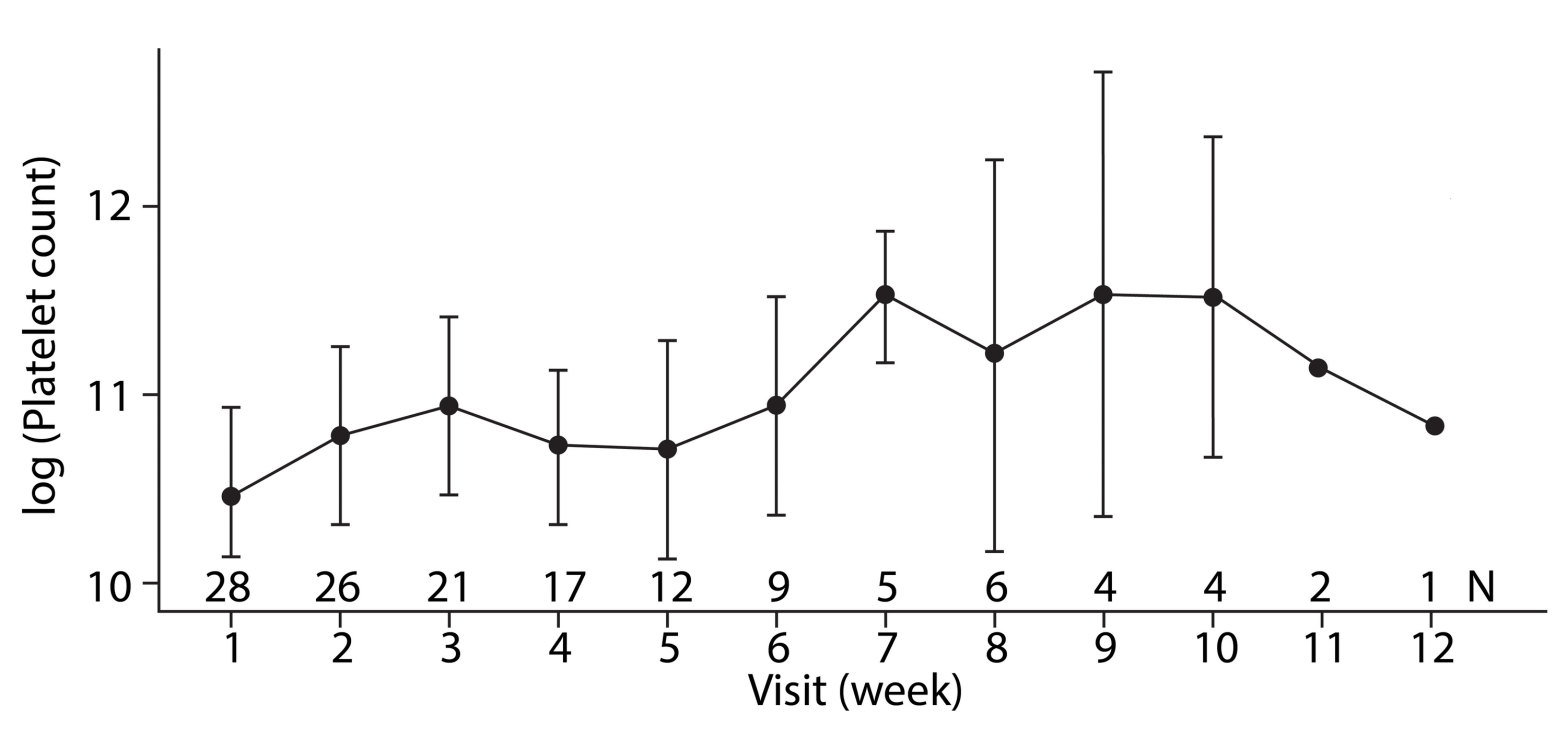
Platelet response over the time with romiplostim

A platelet response at week 10 was achieved in 85.7% (n = 24/28) of the patients (Figure 2).

**Figure 3:**
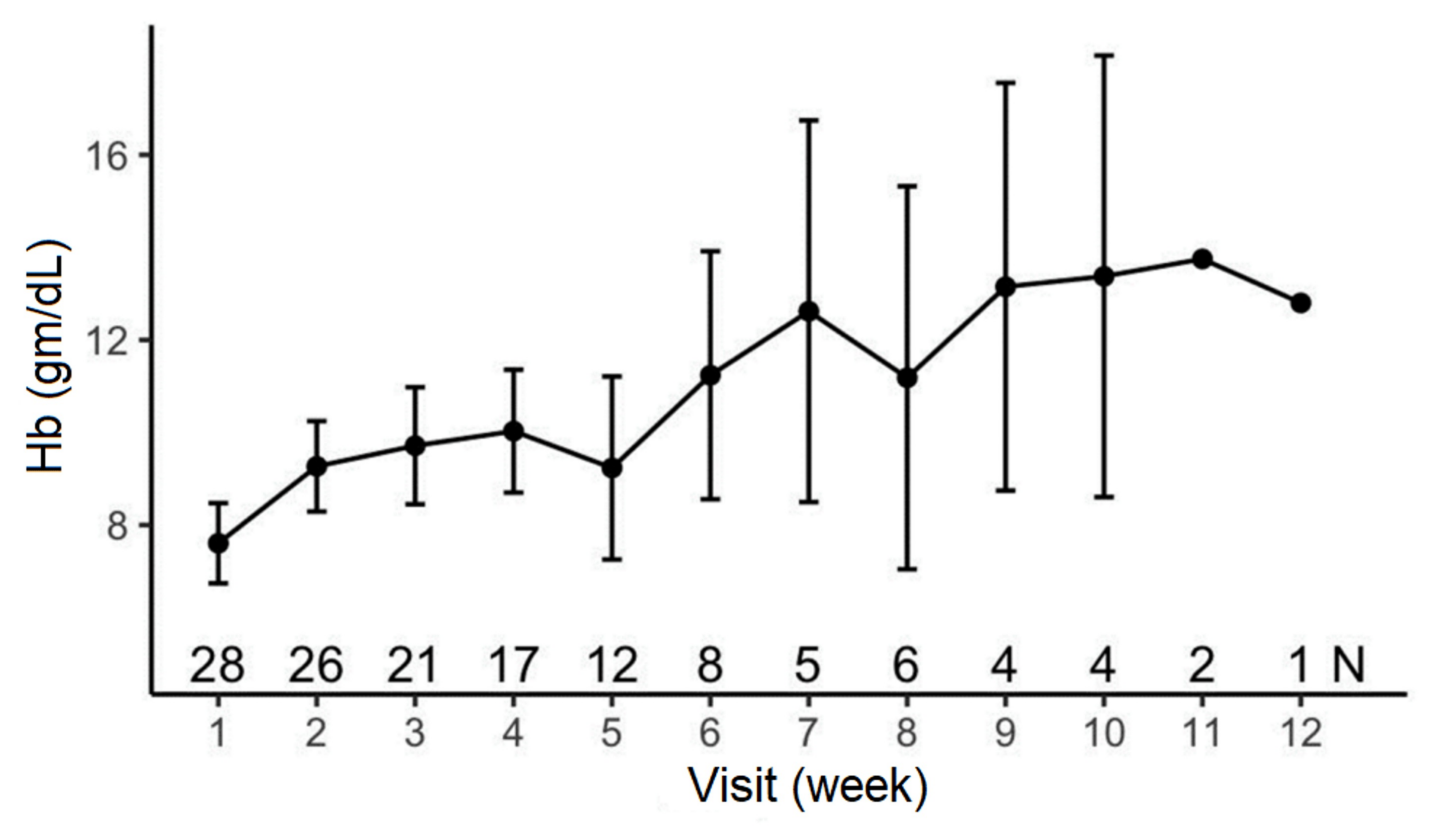
Hemoglobin levels over the study time with romiplostim Hb: Hemoglobin

An increase in platelet count was observed in 24 patients between weeks one and 10. The mean platelet count increased from 70,089/mm³ to 122,000/mm³ (95% CI, p < 0.05) (Figure 2, Table 3). A significant improvement was seen in Hb levels in 85.7% (n = 24/28) of patients by 11 weeks (Figure 3). A gradual increase in Hb levels was seen through weeks one to seven with a decline at week eight, which again spiked at week nine, thereby remaining almost stable.

**Table 3:** Distribution of platelet count, hemoglobin, and total leucocyte count across baseline and follow-up Hb: Hemoglobin; TLC: Total leucocyte count; NA: Not available

Patient visits	N (%)	Hb (g/dL)		TLC (mm^3^)		Platelet (mm^3^)	
		Mean	SD	Mean	SD	Mean	SD
Visit 1	28 (100%)	7.61	2.24	4.20×10^3^	3.32×10^3^	70.09×10^3^	100.20×10^3^
Visit 2	27 (96.4%)	8.49	2.23	4.64×10^3^	3.14×10^3^	129.59×10^3^	170.97×10^3^
Visit 3	26 (92.8%)	9.27	2.42	4.82×10^3^	1.95×10^3^	87.63×10^3^	95.40×10^3^
Visit 4	21 (75%)	9.71	2.78	4.35×10^3^	1.59×10^3^	85.05×10^3^	70.52×10^3^
Visit 5	17 (60.7%)	10.03	2.58	4.86×10^3^	2.87×10^3^	62.18×10^3^	52.52×10^3^
Visit 6	12 (42.8%)	9.23	3.11	4.25×10^3^	2.01×10^3^	63.67×10^3^	51.07×10^3^
Visit 7	8 (28.5%)	11.24	3.2	3.81×10^3^	1.17×10^3^	70.22×10^3^	37.39×10^3^
Visit 8	5 (17.8%)	12.62	3.32	4.60×10^3^	1.04×10^3^	104.20×10^3^	30.91×10^3^
Visit 9	6 (21.4%)	11.18	3.94	4.03×10^3^	1.13×10^3^	98.33×10^3^	53.71×10^3^
Visit 10	4 (14.2%)	13.15	2.77	4.46×10^3^	1.29×10^3^	122.00×10^3^	70.91×10^3^
Visit 11	4 (14.2%)	13.38	3	3.90×10^3^	1.74×10^3^	112.00×10^3^	58.25×10^3^
Visit 12	2 (7.14%)	13.75	2.05	26.75×10^3^	34.29×10^3^	72.00×10^3^	28.28×10^3^
Visit 13	1 (3.5%)	12.8	NA	3.10×10^3^		51.00×10^3^	NA

**Figure 4:**
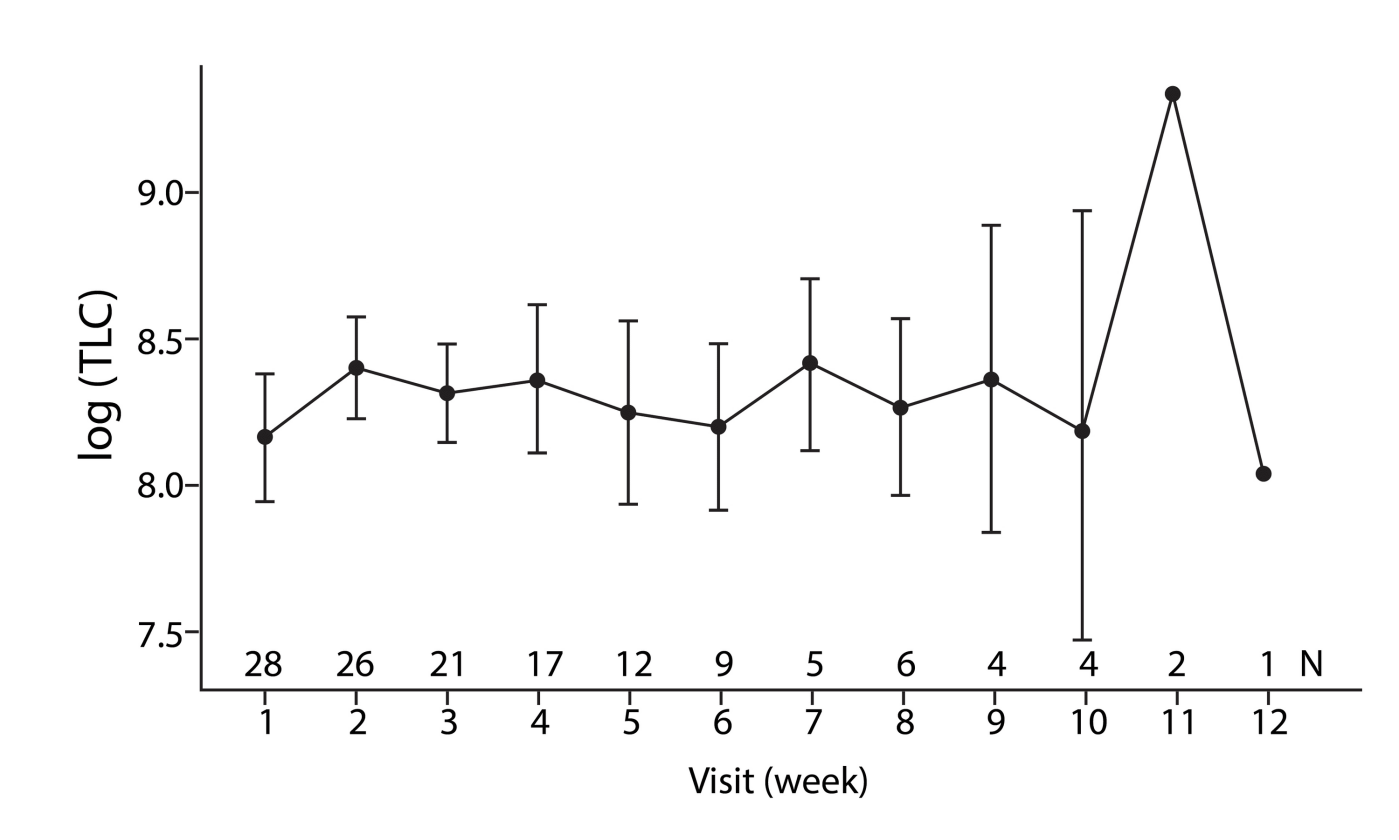
Total leukocyte count (TLC) over the study period with romiplostim TLC: Total leucocyte count

The mean Hb level increased from 7.61 gm/L to 13.38 gm/L (95% CI, p < 0.001). The TLC improved by week 10 in 85.7% (n = 24/28) of patients (Figure 4, Table 3). The mean TLC increased from 4,196/mm^3^ to 4,455/mm^3^ (95% CI, p < 0.04). The remaining four patients showed improvement in all the hematological parameters by 12 weeks.

The graph shows a gradual increase in TLC values through weeks 1-10, with a marked spike at week 11, followed by a decline by week 12.

Safety

Romiplostim was discontinued for one patient after a week of treatment due to a severe headache. In another case, it was halted after the second week because of the seizures. Additionally, a patient chose to cease romiplostim therapy in the third week, but the symptoms were not specified. Furthermore, another patient discontinued romiplostim after three months of treatment; however, no serious adverse events were reported throughout the study period. A total of four patients (14.28%) out of 28 experienced adverse events.

## Discussion

In this retrospective study, all the patients with refractory AA showed significant improvements in platelet count, Hb level, and TLC with romiplostim therapy. Any hematological response was achieved in 85.7% of patients between weeks 10 and 11. The phase II/III study of romiplostim in refractory AA reported a response rate of 84% for any hematological parameter at week 27 [11]. An Indian study in patients with AA having received romiplostim with ATG and cyclosporine showed a hematological response at weeks 12 and 24 and was observed in 50% and 66.67%, respectively [5].

Lee et al. [3] conducted a multi-center phase II study with a randomized, parallel, dose-finding phase (eight weeks), followed by a long-term open-label extension in adult patients with AA refractory to IST. Out of 35 patients, all 10 patients who received romiplostim (10 μg/kg) showed platelet responses, with 30% showing erythroid responses and 60% showing neutrophil responses during the first eight weeks. By week nine, platelet response was achieved in a dose-dependent manner in 30% of patients, with responses in seven of 10 who received romiplostim (10 μg/kg) and three of nine who received romiplostim 6 μg/kg. Platelet responses at weeks 105 and 157 were maintained in 10 patients who received 3-20 μg/kg once weekly, and erythroid and neutrophil responses were observed in nine and five patients, respectively. In five patients, a trilineage response was observed at weeks 53, 105, and 157. Dose tapering was permitted in patients with stable platelet response at weeks 57 and 157. Three patients were able to discontinue romiplostim at 56, 483, and 490 days, respectively [3,12].

Mitani et al. [1] studied the long-term efficacy and safety of romiplostim in refractory AA beyond 52 weeks. During the study follow-up period of 3.5 years, nearly half the patients not achieving platelet or erythrocyte responses to romiplostim at week 52 eventually achieved responses by continuing the administration until the end of the study. This demonstrates the efficacy of prolonged romiplostim administration [1]. Romiplostim was also studied in combination with ATG plus cyclosporine in patients with refractory AA. The hematological response rate was achieved in 76.5% of patients at the end of week 27 [13]. The same study also reported that 87.5% (n = 14/16) of patients achieved transfusion independence [13].

Limitations

The retrospective nature and small study population size are the primary limitations of the study. There is a strong likelihood of bias in the data collection process for the study parameters. The small study population limits the statistical power and generalizability of the results. Despite the smaller population due to the rarity of the disease, the study adds value because it showcases real-world data. Larger, prospective, randomized controlled trials to confirm these findings and the exploration of romiplostim’s impact in combination therapies across diverse population are necessary.

## Conclusions

Romiplostim is efficacious and well-tolerated in patients with refractory AA who were previously treated with immunosuppressive therapy. Similar to the previously published studies, romiplostim, a TPO analog, effectively increases platelet production. Romiplostim can be considered a valuable therapeutic option for Indian patients with refractory AA.
