# Correction: Romiplostim in Aplastic Anemia: A Single-Center Retrospective Study

**DOI:** 10.7759/cureus.c434

**Published:** 2026-05-19

**Authors:** Vijay Ramanan, Ketki Kelkar

**Affiliations:** 1 BMT Department and Clinical Hematology, Yashoda Hematology Clinic, Ruby Hall Clinic, Pune, IND; 2 Hematology, Anjali Diagnostic Laboratory, Genepath Pathology, Mumbai, IND

This article has been corrected to change all dosing of Romiplostim to mcg (micrograms) rather than mg (milligrams).

